# Lunatics in the Good Old Times

**Published:** 1860-08

**Authors:** 


### LUNATICS IN THE GOOD OLD TIMES.

It is impossible for us to imagine the condition of lunatic asylums before the days of Pinel. Dungeons, wet and infected, without light and air, called *loges*, containing a wretched mattress, or some rotten straw strewed on the ground. Human beings, naked or covered with rags, almost always furious, chained to each other, and shut up in these abodes of desolation and misery—actual tombs, out of which they came to be carried to their last dwelling place. Their brutal keepers, selected from those who had been condemned to punishment, treated them like brutes, and used them most barbarously, abusing and ridiculing them, striking them cruelly, and continually fighting with them, throwing them their deficient and coarse food, keeping their water from them when thirsty, and their clothes when cold; exposing them to the jokes of visitors. Such were the inhabitants of Bicetre at that time—wretched beings thought incurable, abandoned by their friends, deprived of all medical treatment, pale, wasted, wallowing in their own filth, groaning under the weight of the chains which tore their wasted limbs, raving under the horrible sufferings to which they were subjected by their inhuman keepers.

Pinel.—When Pinel undertook the management of Bicetre the revolution was at its height. The notorious Couthon presided over the dreaded *Commune* of Paris, and when Pinel came to him for permission to remove the chains from the madmen, went himself the next day to the asylum, fearing lest in such an act there should be some hidden attempt against the Democratic Government. When he saw the madmen, he turned to Pinel and said: "Are you not mad yourself, to wish to deliver these *betes feroces* from their chains?" "No," answered Pinel, "for I am certain that their chains make these wretched people thus violent." "Do as you like," said Couthon. From this time the good work commenced. The following day he removed the chains from fifty, and from thirty more a few days afterwards. The Academy of Medicine has adorned one of its rooms with a picture of this scene of humanity. Shortly after this we are told that Pinel was seized by some of the ruffians of the day, under the pretext of being an aristocrat, and hurried off "*a la lanterne;*" and that his life was saved by an old soldier, one Chevigne, whom he had delivered from his chains in the Bicetre, and who had become his servant.

Pinel states, in letters of his lately published, that he was enabled to live decently on what he obtained by the translation of English works. "At this moment," he says, "I am engaged in the translation of Cullen's Institutes of Medicine, for which I receive one thousand francs. I would give up physic altogether, if it were necessary for me to be running about all day in the streets. I wish for only a very small practice—to see little and observe much."

